# Thirteen Years of Impactful, Minimally Invasive Coronary Surgery: Short- and Long-Term Results for Single and Multi-Vessel Disease †

**DOI:** 10.3390/jcm13030761

**Published:** 2024-01-28

**Authors:** Lilly Ilcheva, Achim Häussler, Magdalena Cholubek, Vasileios Ntinopoulos, Dragan Odavic, Stak Dushaj, Hector Rodriguez Cetina Biefer, Omer Dzemali

**Affiliations:** 1 Department of Cardiac Surgery, University Hospital Zurich, Rämistrasse 100, 8091 Zurich, Switzerland; lilly.ilcheva@usz.ch (L.I.); achim.haeussler@usz.ch (A.H.); magdalena.cholubek@usz.ch (M.C.); vasileios.ntinopoulos@stadtspital.ch (V.N.); dragan.odavic@stadtspital.ch (D.O.); stak.dushaj@usz.ch (S.D.); hector.rodriguez@usz.ch (H.R.C.B.); 2 Department of Cardiac Surgery, Zurich City Hospital—Triemli, Birmensdorferstrasse 497, 8055 Zurich, Switzerland

**Keywords:** cardiac surgery, coronary artery bypass graft, MIDCAB, MICS, MICSCABG

## Abstract

Objectives: Minimally invasive coronary surgery (MICS) via lateral thoracotomy is a less invasive alternative to the traditional median full sternotomy approach for coronary surgery. This study investigates its effectiveness for short- and long-term revascularization in cases of single and multi-vessel diseases. Methods: A thorough examination was performed on the databases of two cardiac surgery programs, focusing on patients who underwent minimally invasive coronary bypass grafting procedures between 2010 and 2023. The study involved patients who underwent either minimally invasive direct coronary artery bypass grafting (MIDCAB) for the revascularization of left anterior descending (LAD) artery stenosis or minimally invasive multi-vessel coronary artery bypass grafting (MICSCABG). Our assessment criteria included in-hospital mortality, long-term mortality, and freedom from reoperations due to failed aortocoronary bypass grafts post-surgery. Additionally, we evaluated significant in-hospital complications as secondary endpoints. Results: A total of 315 consecutive patients were identified between 2010 and 2023 (MIDCAB 271 vs. MICSCABG 44). Conversion to median sternotomy (MS) occurred in eight patients (2.5%). The 30-day all-cause mortality was 1.3% (n = 4). Postoperative AF was the most common complication postoperatively (n = 26, 8.5%). Five patients were reoperated for bleeding (1.6%), and myocardial infarction (MI) happened in four patients (1.3%). The mean follow-up time was six years (±4 years). All-cause mortality was 10.3% (n = 30), with only five (1.7%) patients having a confirmed cardiac cause. The reoperation rate due to graft failure or the progression of aortocoronary disease was 1.4% (n = 4). Conclusions: Despite the complexity of the MICS approach, the results of our study support the safety and effectiveness of this procedure with low rates of mortality, morbidity, and conversion for both single and multi-vessel bypass surgeries. These results underscore further the necessity to implement such programs to benefit patients.

## 1. Introduction 

After performing the first successful open-heart surgery on cardiopulmonary bypass (CPB) in 1953, median sternotomy (MS) became the gold standard surgical approach in cardiac surgery due to its safety and ease of reproducibility [1]. Nevertheless, MS and inherited invasiveness are associated with complications such as substantial bleeding and requiring blood transfusion, infections, and an extended recovery period to achieve optimal physical activity [2]. The landscape of conventional cardiac surgery began to change in 1967, with Kolesov performing the first minimally invasive coronary surgery (MICS) on the beating heart via a left thoracotomy [3]. In the early 1990s, surgeons such as Benetti, Calafiore, Subramanian, and Boonstra conducted the first series of MICS [4,5,6,7]. They demonstrated the durability and safety of minimally invasive direct coronary artery bypass (MIDCAB) in cases of left anterior descending artery (LAD) stenosis (0–3.8% in-hospital mortality, postoperative graft patency rate 92% to 100%, 93% freedom of cardiac events in the first 30 days, and 92.2% after mean follow up of 5.6 months). At the beginning of MICS, a significant challenge was accessing the coronary arteries and performing precise anastomoses through a minimal incision. These challenges were overcome by developing specialized retractors and stabilizers, which improved visualization of the internal thoracic arteries and the ascending aorta [8,9]. Additionally, a cardiac apical positioner was implemented to manipulate the heart and enhance exposure to the coronary territories [10,11]. The graft-to-coronary anastomosis was stabilized using an epicardial stabilizer [12]. To ensure optimal outcomes, the anesthesiology team also played a vital role in managing intrathoracic and intracardiac pressure [13]. In various studies, MICS demonstrated comparable results to coronary artery bypass surgery (CABG) through MS, particularly regarding long-term patency rates and other well-known complications [2,14,15,16,17,18]. MICS has been associated with many advantageous outcomes, including decreased postoperative pain, enhanced cosmetic results, reduced surgical site infection rates, minimized surgical trauma, fewer blood transfusion requirements, and faster recovery and hospital discharge times [2,14,15,16,17,18]. Numerous studies have indicated that surgical coronary revascularization is superior to percutaneous coronary intervention (PCI) in both the short-term and long-term [19,20,21,22].

Despite the well-documented benefits of MICS, there is a noticeable reluctance among medical centers to adopt this technique [16]. This can largely be attributed to the technical demands of such procedures. Although single LAD stenosis treatment with MIDCAB is a relatively more accepted procedure, minimally invasive multi-vessel coronary artery bypass (MICSCAB) is less frequently performed [16]. Furthermore, despite the longevity of MICS, data scarcity remains a concern.

The present study comprehensively analyzes the short- and long-term outcomes of inpatients diagnosed with single and multiple-vessel diseases. The study aims to furnish descriptive data to corroborate the effectiveness of MICS, thereby contributing to the existing literature in this domain.

## 2. Materials and Methods 

### 2.1. Patients

Retrospective data analysis was carried out on all consecutive patients who underwent either single (MIDCAB) or multi-vessel (MICSCABG) minimally invasive coronary artery bypass surgery at the University Hospital of Zurich and the City Hospital of Zurich Triemli between January 2010 and September 2023. Only patients who did not provide general research consent were excluded from this study.

The data collected included demographics (age, gender), comorbidities (history of previous cardiac surgeries and interventions, record of last myocardial infarct (MI), chronic obstructive pulmonary disease (COPD), chronic renal failure, peripheral artery or cerebrovascular disease), New York Heart Association (NYHA) class, Canadian Cardiovascular Society (CCS) Angina Score, European System for Cardiac Operative Risk Evaluation II (EuroSCORE), operative setting (elective or urgent, with ‘urgent surgery’ explicitly referring to cases requiring an operation within the same hospitalization period), preoperative and postoperative left ventricular ejection fraction (LVEF), the number of diseased vessels, pre-and postoperative serum creatinine levels, the usage of CPB, the duration of surgery, the type of grafts, target vessels, the number of distal anastomoses performed, the rate of conversion to sternotomy, Intensive Care Unit (ICU) and hospitalization duration, blood product transfusions, in-hospital postoperative complications, and in-hospital mortality rate. Additional data regarding survival and interventions during the follow-up period were gathered via telephone interviews with the treating cardiologists and general practitioners. 

The Local Ethics Committee approved the study. 

### 2.2. Surgical Strategy and Approach 

The treatment strategy for all patients was determined after an interdisciplinary discussion by the HEART-TEAM based on the individual coronary anatomy, the complexity of the coronary lesions, and the patient’s conditions. MICSCABG with a maximum of three target vessels was aimed for in cases of multivessel disease. If hybrid revascularization was indicated, it was either a concurrent or staged procedure.

All operations were conducted using a left mini-thoracotomy approach. In short, the left mini-thoracotomy involved making a 5 cm incision beneath the mammary region on the left side of the anterior-lateral chest. Access to the thoracic cavity was achieved diagonally through the fourth or fifth intercostal space. CPB is only employed as a bailout strategy in cases of hemodynamic instability. If CPB was used, arterial and venous cannulations were performed percutaneously via the femoral artery and vein, subsequently positioning the cannula with the help of transesophageal guidance. A Bretschneider cardioplegic solution served as myocardial protection. Depending on the surgeon performing the procedure, a rib-spreading retractor was used to harvest the LIMA, which was either fully skeletonized or semi-skeletonized. When necessary, grafts from the great saphenous vein (GSV) or radial artery (RA) were extracted via endoscopic or traditional open methods. The LIMA-LAD procedure was first performed using a stabilizer, after which other coronary targets were addressed sequentially using the RA. If more than one revascularization was required, a Y-shaped construct was created before performing the distal anastomosis. The pericardium was closed in all patients after performing the anastomosis, and the pulsatile flow was secured. A singular tube was positioned on the left side of the chest. The ribs were approximated with one biodegradable. The incision was closed in layers in a standard manner. All patients received local anesthesia after surgery at the thoracotomy site to minimize post-surgical pain. Multiple surgeons were involved in this series, and the operating surgeon determined conduit selection and configuration.

### 2.3. Statistical Analysis

We analyzed preoperative, intra-operative, postoperative, and follow-up data using SPSS version 29.0. The normality of continuous variables within the dataset was assessed using the Kolmogorov–Smirnov and Shapiro–Wilk tests. Continuous variables were expressed as mean ± standard deviation or median (interquartile range (IQR)) as appropriate. Subsequently, continuous variables were analyzed via the Mann–Whitney U test. Categorical variables were analyzed employing the chi-square test. A *p*-value of <0.05 was considered indicative of statistical significance. Survival curves were constructed using the Kaplan–Meier method and analyzed using the log-rank test.

The study’s primary endpoints were in-hospital and long-term mortality, defined as mortality during the follow-up period and freedom from reoperations due to failed aortocoronary bypass grafts during follow up. Furthermore, survival analysis was conducted to determine whether there were differences in survival and reoperation rates among patients undergoing MIDCAB and MICSCAB, as well as between elective and urgent cases.

Secondary endpoints included major in-hospital complications such as postoperative bleeding and the need for a rethoracotomy, stroke, myocardial infarction (MI), declines in renal function and LVEF postoperatively, the rate of new atrial fibrillation (AF), and the frequency of pacemaker implantations and postoperative wound infections requiring surgical intervention during the patient’s initial hospitalization period. 

## 3. Results

We identified 315 consecutive patients in our databases during the study period. The average age of the study participants was 64 ± 11 years. Among them, 19 patients (6%) were aged 80 or older, while 31 patients (9.8%) fell within the age range of 75 to 79. Female patients constituted a substantial minority, comprising 53 (16.8%) of the total cohort. A total of 182 patients (57.8%) were diagnosed with single-vessel disease, 79 patients (25.1%) with two-vessel disease, and 54 patients (17.1%) with three-vessel disease. Furthermore, 118 patients (37.5%) had a history of PCI, and 64 patients (20.3) experienced an MI within 30 days before the operation. Upon admission, 54 patients (17.1%) were diagnosed with CCS stage 3, 22 patients (7%) with CCS stage 4, and 42 patients (13.3%) required urgent surgery. The median preoperative LVEF was 60%, with a range of 50% to 64%, and the median EuroSCORE II was 1.2 (0.8–2.2). A comprehensive overview of the preoperative characteristics of the patients is provided in Table 1.

**Figure 1:**
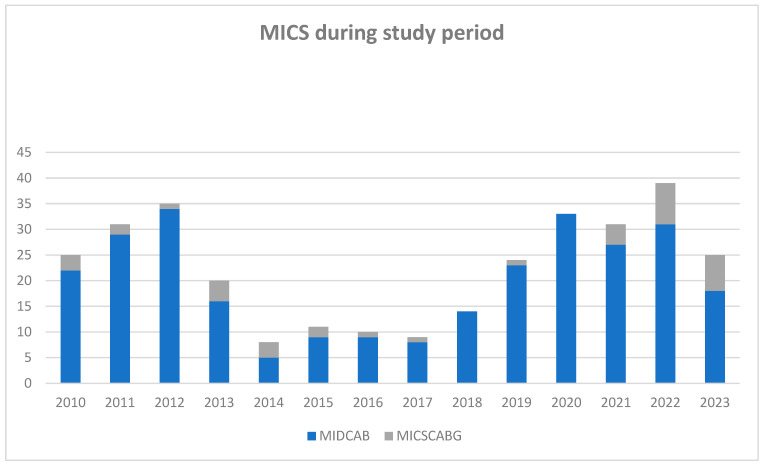
Number of minimally invasive direct coronary artery bypass grafting operations during the study period. MICS: minimally invasive coronary surgery during the follow up (n = 315); MIDCAB: minimally invasive direct coronary artery bypass grafting (n = 271); and MICSCABG: minimally invasive multi-vessel coronary artery bypass (n = 44).

Operative details are comprehensively outlined in Table 2. Most patients underwent MIDCAB (271 patients, 86%), while the remaining 44 (13%) received MICSCABG. As depicted in Figure 1, the case volume experienced fluctuations during the study period. The median duration of surgery was recorded at 139 min (115–172 min). The LIMA was the most frequently utilized graft, implemented in 313 patients (99.4%). Additionally, the great saphenous vein (GSV) was used in 17 patients (5.4%) and the radial artery (RA) in 16 (5.1%) cases. Revascularization predominantly targeted LAD, performed in 301 cases (97.1%). Subsequent interventions involved the diagonal branches in 27 patients (8.6%), the circumflex artery (RCx) in 16 patients (5.1%), and the right coronary artery (RCA) in 3 cases (1%). CPB was used in 12 patients (3.9%). Intraoperative median graft flow was measured at 24 mL/min (17–35 mL/min) and a median pulsatility index (PI) of 1.9 (1.5–2.6). Conversion to sternotomy was necessitated in eight cases (2.5%) due to various reasons, including lung adhesions, an intramyocardial course of the LAD, hemodynamic instability during the procedure, inadequate exposure due to an elongated aorta, and technical issues with anastomosis. Two patients (25%) of this group died within the first 30 days postoperatively: one patient (12.5%) because of cardiogenic and distributive shock, and one patient (12.5%) because of disseminated intravascular coagulation and intraoperative pulmonary embolism and excessive extracorporeal membrane oxygenation. Due to the intended analysis of this study, converted patients were excluded from follow up.

### 3.1. In-Hospital Outcomes

In-hospital outcomes are shown in Table 3.

The highest rate of in-hospital complications was observed in the form of new onset of AF, which was observed in 26 patients (8.5%). Five patients (1.6%) suffered postoperative MI, and a further five patients (3.2%) underwent rethoracotomy due to bleeding. Additionally, postoperative wound infections of the thoracotomy occurred in four (1.3%) of the subjects, and one patient (0.3%) developed a postoperative atrioventricular (AV) block that necessitated the implantation of a permanent pacemaker. Notably, no cerebrovascular events or acute renal failure required renal replacement therapy within the follow-up period. The median length of stay in the intensive care unit (ICU) was two days (IQR: 1–3 days), with a median hospital stay of 8 days (IQR: 7–9 days).

### 3.2. 30-Day Mortality

**Figure 2:**
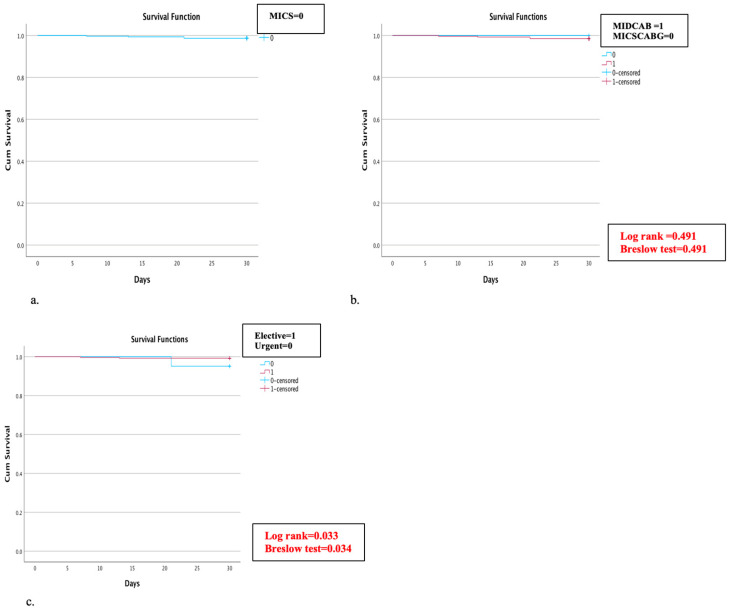
(**a**–**c**) Kaplan–Meier curve for 30-day survival rate: (**a**) 30-day survival rate in all MICS patients was estimated at 98.7%; (**b**) subgroup analysis of the 30-day survival in the minimally invasive direct coronary artery bypass grafting (MIDCAB) and minimally invasive multi-vessel coronary artery bypass (MICSCABG) patients; and (**c**) subgroup analysis of 30-day mortality in elective and urgent cases.

Four patients (1.3%) died within 30 days after surgery, and the survival rate for the entire cohort was 98.7% (Figure 2a). Three patients (1%) died due to cardiogenic shock, and one patient (0.3%) of septic shock due to a preexisting graft infection of the abdominal aorta. Of note, no significant difference in the 30-day survival rate between the MIDCAB (98.5% 30-day survival rate) and MICSCABG patients (100% 30-day survival rate) (Log-rank = 0.491, Breslow test = 0.491) was observed (Figure 2b). Still, there was a significant difference between the elective and urgent patient groups (99.2% vs. 95.1% 30-day survival rate) (Log rank = 0.033, Breslow test = 0.034) (Figure 2c).

## 4. Follow-Up Data 

**Figure 3:**
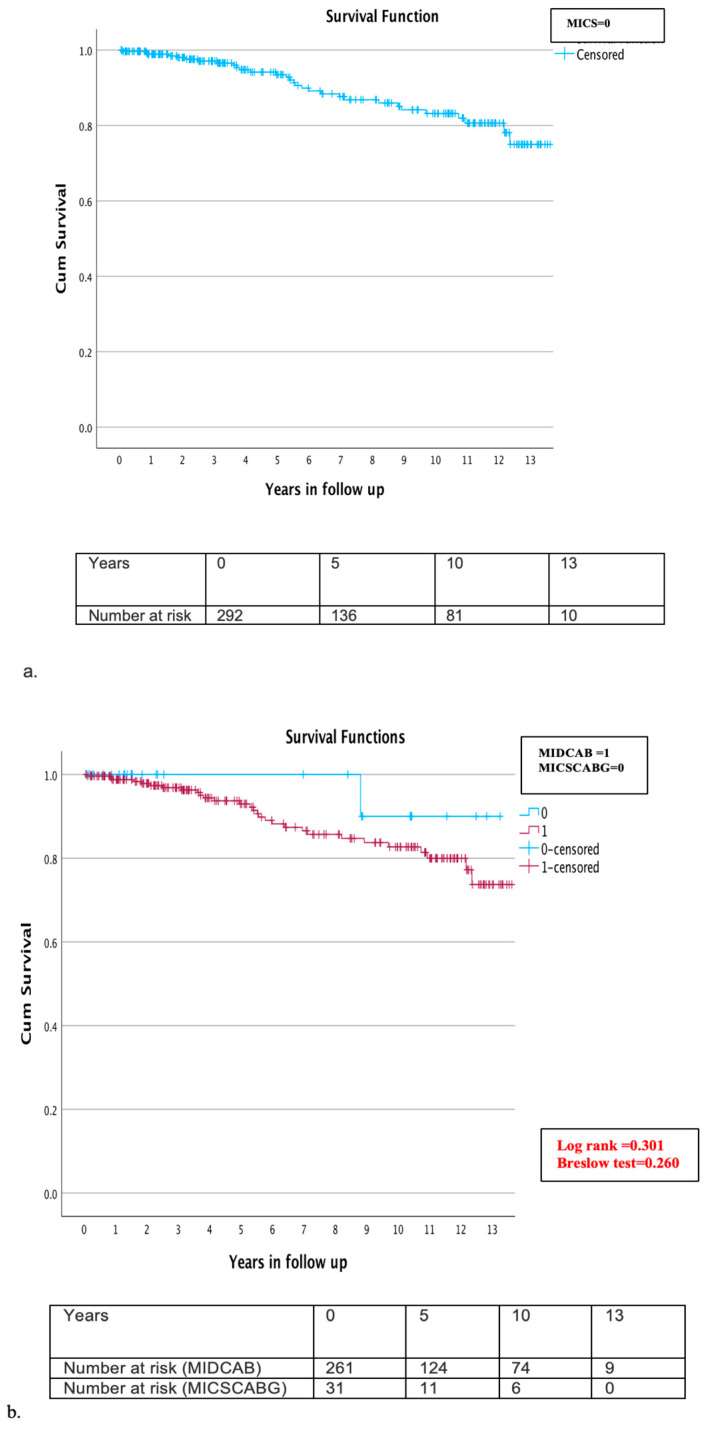

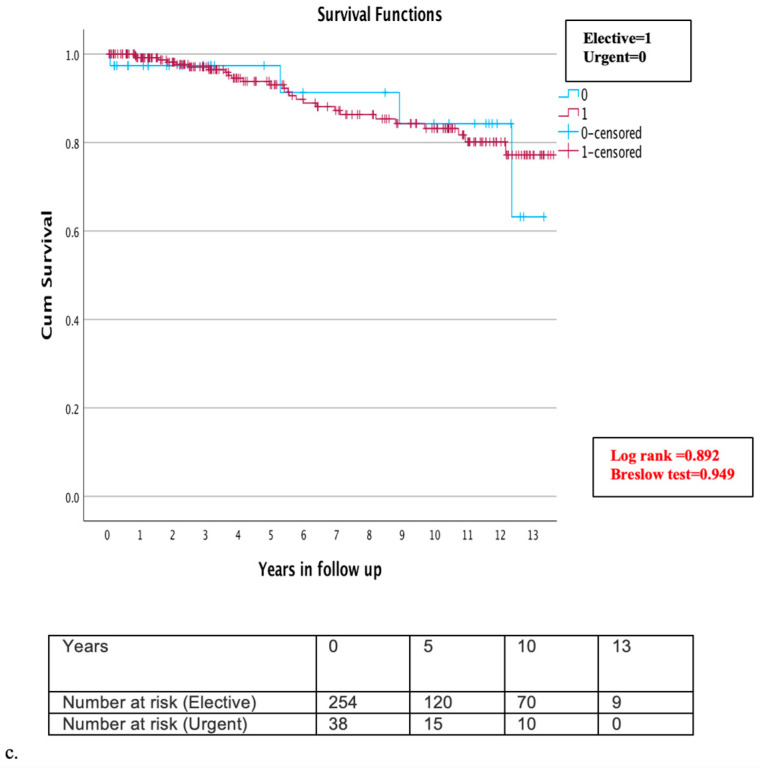
(**a**–**c**) Kaplan–Meier overall survival curve during the follow up (**a**) overall survival at 5, 10, and 13 years for all MICS patients was 95.9%, 91.8%, and 89.7% (**b**) subgroup analysis of the MIDCAB and MICSCABG patients during the follow up: survival rate in the MIDCAB group at 5, 10, and 13 years was 95.4%, 90.4%, and 88.9%, and in the MICSCABG group, 100%, 96.8%, and 96.8%, respectively; and (**c**) subgroup analysis of the elective and urgent cases during follow up: survival rate in the elective group at 5, 10, and 13 years was 95.3%, 90.6%, 89.8%, and in the urgent group, 97.4%, 92.1%, and 89.5%, respectively.

The follow-up completion rate was 95.4% (n = 292). The mean follow-up time was six years (±4 years). A total of eleven patients (3.6%) were lost during follow up. Throughout the follow-up period, mortality was observed in 30 patients (10.3%), yielding an overall survival rate of 5,10, and 13 years of 95.9%, 91.8%, and 89.7%, respectively (Figure 3a). A detailed examination of mortality causes revealed that cardiac-related deaths were verified in only five cases (1.7%), while the cause of death remained unknown in 12 cases (4.1%). Comparative subgroup evaluation for long-term morality was conducted between the MIDCAB (261 patients) and MICSCABG (31 patients) and between elective (254 patients) and urgent (38 cases). During the long-term follow up, 29 patients (11.1%) of the MIDCAB and one patient (3.2%) of the MICSCABG group died. The estimated survival rate in the MIDCAB group at 5, 10, and 13 years was 95.4%, 90.4%, and 88.9%, and in the MICSCABG group, 100%, 96.8%, and 96.8%, respectively (Figure 3b,c). No significant difference was detected in either group (Log rank = 0.301, Breslow test = 0.260) (Figure 3b,c). Twenty-six patients (10.2%) died in the elective group and four (10.5%) in the urgent group. The survival rates in the elective and urgent groups at 5, 10, and 13 years were 95.3%, 90.6%, and 89.8% and 97.4%, 92.1%, and 89.5%, respectively, without significant difference (Log rank = 0.892, Breslow test = 0.949) (Figure 3b,c). During the follow up, four patients (1.4%) required re-admission and cardiac reoperation due to graft failure or the progression of aortocoronary disease and underwent uneventful reoperation through MS. Cardiac reoperation rate in the MIDCAB group was 1.5% and in the MICSCABG, 0%.

During the follow up, two patients (0.7%) were readmitted (5 months postoperatively and 7 months postoperatively) due to pulmonary herniation and were treated with reoperation. Another two patients (0.7%) were readmitted (21 days postoperatively and 26 days postoperatively) for purulent wound infections. They were successfully treated with IV antibiotics, vacuum-assisted closure (VAC), and suture closure, without further complications.

## 5. Discussion

Median full sternotomy offers optimal visibility for accessing all areas of the heart. Moreover, it enables the assessment of each coronary artery via direct external examination and palpation to identify a suitable distal target location. Consequently, this approach, combined with CPB and cardiac arrest, has become the predominant surgical technique for most coronary artery bypass grafting (CABG) procedures worldwide [23,24].

Due to excellent long-term results compared to PCI, CABG remains the gold standard for myocardial revascularization. However, CABG via MS predisposes the patient to the risk of superficial or deep sternal wound infections, mediastinitis, and sternal instability, threatening patients’ lives and quality of life; thus, MICS remains a valid alternative to offer patients with optimal surgical therapy with fewer complications [16,25,26,27,28,29]. MICS was first performed in the 1960s and remains a technically demanding procedure. Despite technological advancements over the past decades, very few centers worldwide practice MICS, either MIDCAB or MICSCABG [16,30].

The current experience dating over 13 years presented in this study corroborates that MICS is a safe and feasible method with favorable short- and long-term outcomes. Our data shows a 30-day mortality rate of 1.3% for the entire cohort. Furthermore, the long-term survival rate for the whole cohort at 5, 10, and 13 years was 95.9%, 91.8%, and 89.7%; more importantly, our cohort showed only 1.7% mortality due to a cardiac cause during the entire follow up. The results of our study confirmed that MICS offers a safe and effective method for revascularization. The initial reports of MIDCAB by Benetti, Calafiore, Subramanian, and Boonstra demonstrated an in-hospital mortality range between 0% and 3.8% [4,5,6,7]. Recently, Manuel et al. published 20-year outcomes of 271 patients undergoing MICS and reported 5-, 10-, 15-, and 20-year survival rates of 91.9%, 84.7%, 71.3%, and 56.5% [31]. Bonatti et al.’s review of the MICS in the last 25 years reported an in-hospital rate of 99% and a 5-year survival rate of 91% [16]. Davierwala et al. analyzed 2667 patients over 20 years and reported, in-hospital, a 5-, 10-, 15- and 20-year survival at 99.1%, 88%, 77.7%, 66.1%, and 55.6% [29]. Repossini et al. reported 30-day, 5-, 10-, and 15-year survival rates of 99.2%, 87.1%, 84.3%, and 79.8% in a single-center experience with 1060 patients [2]. The outcomes of our analysis reveal comparable short-term mortality rates and even superior long-term results. This underscores the initial belief that minimizing the surgical incision size does not necessitate compromising the procedure’s safety.

Our findings should be evaluated in the context of MICS and compared to the traditional CABG with or without CPB. A recent report from The Society of Thoracic Surgery Adult Cardiac Surgery Database (2020–2021), analyzing 137,856 (2020) and 153,208 (2021) CABG patients, observed all-cause mortality rates during hospitalization of 2.7% and 2.5%, respectively [32]. The short-term mortality data from MICS reported by Bonatti, Davierwala, and Repossini et al., combined with our findings, show mortality outcomes that are comparable or, in some instances, superior to those of conventional aortocoronary bypass surgery for coronary revascularization [2,16,29].

Long-term all-cause mortality in our group was 10.3%, and notably, only 1.7% experienced cardiac death as the cause. The CORONARY trial, a large randomized trial comparing on-pump and off-pump CABG, reported a 5-year mortality of 13.5% in the on-pump group and 14.6% in the off-pump CABG arm [33]. The analysis of Chikwe et al. demonstrated 10-year rates of mortality after off-pump and on-pump surgery at 33.4% and 29.6%, respectively [17]. Our study’s findings indicate that MICS’s short- and long-term mortality outcomes are comparable and even slightly superior to those of the leading trials in aortocoronary bypass surgery, whether on- or off-pump. These results highlight the potential of MICS as a safe and effective alternative to traditional CABG surgery. 

Emergent conversion to MS due to bleeding, lack of exposure to the surgical field, intramyocardial course of LAD, hemodynamic instability, rethoracotomy because of bleeding, and postoperative MI are the main concerns regarding MICS. Our study compares favorably with systematic reviews of MIDCAB short and mid-term results reporting the conversion rate to sternotomy (CPB of 0% to 7%) and perioperative infarction rates of 0% to 3.1% [28,34]. Of note, we reported a 2.5% conversion rate and a 1.6% infarction rate. Wound infections such as deep sternal wound infections and mediastinitis present serious complications after cardiac surgery via MS, vary between 1.3 to 12.8% according to Jonkers et al., and are related to higher mortality [35,36]. One advantage of performing sternal-sparing revascularization via lateral thoracotomy is the reduced incidence of associated complications. Wound infections in the MICS vary between 0.3% and 2.2% [16,25,26,27,28,29]. A case-matched study between MICS and OPCAB via sternotomy by Lapierre et al. demonstrated slightly lower wound infection in the MICS group (0–4%) [37]. Our analysis revealed a 1.3% wound infection rate, comparable to the previous results in the literature. 

Multi-vessel revascularization via MICS continues to pose a challenge for many surgeons, including those experienced in minimally invasive single-vessel revascularization. Bonatti et al. documented a 23.1% rate of MICSCABG in their comprehensive 25-year review of 5226 cases globally [16]. Kyaruzi et al. observed a 1% in-hospital mortality rate in a recent cohort of 100 patients undergoing MICSCABG, with no reported reoperations for graft failure [38]. Une et al. and Babliak et al. conducted retrospective analyses of 210 and 229 cases of MICSCABG, respectively, reporting zero in-hospital mortality rates [39,40]. Rodriguez et al. also reported an absence of short-term mortality and 97.4% freedom from major adverse cardiac and cerebrovascular events at a mean follow-up time of 2.8 years in their initial cohort of 306 patients undergoing MICSCABG [27]. In our study, the cohort of 44 patients who underwent multi-vessel revascularization via MICSCABG demonstrated a 100% survival rate at both the 30-day and five-year benchmarks, with a maintained rate of 96.8% at the 10- and 13-year benchmarks. Notably, there were no instances necessitating reoperation for graft failure during the follow-up period. While our cohort was relatively small compared to more extensive retrospective studies, the outcomes are consistent, indicating that multi-vessel revascularization can achieve sustainable results despite inherent technical challenges. However, the scarcity of long-term data on MICSCABG underscores the need for further research. Consequently, randomized controlled trials (RCTs) are essential to thoroughly evaluate the benefits and potential limitations. 

Additionally, we conducted survival analyses to discern differences in patient outcomes following elective (273 patients, 86.7%) versus urgent (42 patients, 13.3%) MICS procedures. The 30-day mortality rates significantly favored the elective group, which exhibited a 99.2% survival rate compared to 95.1% in the urgent group (Log-rank test *p* = 0.033, Breslow test *p* = 0.034); nevertheless, a 95.1% survival in the urgent group is in our opinion a still remarkable result. Of note, this differential did not extend into the long-term follow up, with survival rates converging at 5, 10, and 13 years to 95.3%, 90.6%, and 89.8% for the elective group, and 97.4%, 92.1%, and 89.5% for the urgent group, respectively. The need for more focused research on the long-term outcomes of urgent or emergency MICS is notable. Nonetheless, our analysis indicates that the long-term survival post-urgent MICS is not significantly different from elective procedures. These findings suggest that the urgency of the procedure should be independent of the use of MICS, affirming its safety and efficacy in diverse clinical scenarios [2,16,29,31]. 

Of note, we observed a 1.4% rate of reoperation for failed bypass grafts during the entire follow up, which, even compared to standard CABG with sternotomy, was remarkably low (between 1.8%–2.2% in the USA, according to the analysis of Elbadawi [41]). A recent research by Gaudino et al. pooled individual patient data from randomized clinical trials comparing OPCAB and on-pump CABG [42,43,44]. This study, focusing on graft patency confirmed through imaging, reported a graft failure rate of 16.6% and an MI rate of 1.9%, measured at a median imaging time of 1.02 years [42,43,44]. Long-term results by Buxton, Goldman, and Gaudino et al. reported 10-year graft failure rates of 39–50% of SVG, 5–15% of LIMA, 20–25% of RIVA, and 9–15% of RA [42,43,44,45,46]. Their findings might contradict the current belief that reduced visualization, space, and heart movement might compromise the quality of a bypass anastomosis.

In terms of morbidity and perioperative complication, it must be taken into consideration that the avoidance of MS and CPB suggests absence or reduced incidence of postoperative stroke and low mortality, reduced rates of postoperative AF, fast-trackable patients, a decreased need for red blood cell transfusions, fewer infections, and shorter ICU and hospital stays after CABG [2,14,25,29,31,47]. Despite the technical complexity of MICS and the reduced exposure of the whole heart, our study demonstrated low in-hospital rates of postoperative MI (1.6%) and reoperations due to bleeding (1.6%). Compared to some of the most extensive meta-analyses of off-pump CABG and on-pump CABG, these findings demonstrated significantly lower rates of these complications [18,48]. In a meta-analysis by Afilalo et al., postoperative MI rates were 3.4% in the off-pump group and 3.9% in the on-pump group. Postoperative stroke rates were 1.4% in the off-pump group and 2.1% in the on-pump group [18]. Puskas et al. reported rates of 2.3% and 2.6% for postoperative bleeding revision in off-pump and on-pump coronary bypass surgery when evaluating 19,101 patients in 102 prospective randomized studies reviewed at the International Society for Minimally Invasive Cardiothoracic Surgery (ISMICS) consensus conference [48].

Furthermore, postoperative AF is the most prevalent cardiac complication occurring postoperatively, affecting approximately 30% of cardiac surgery patients, as indicated by the Framingham Heart Study [49]. Postoperative AF is an indicator of both short-term and long-term cardiovascular problems, such as stroke, infarction, thromboembolism, and cardiac arrest. It may also require reoperation due to internal bleeding, and patients with postoperative AF have a two-fold more significant risk of all-cause 30-day and six-month mortality [50]. In the literature, the documented occurrence of postoperative AF after MICS is between 1.7% and 24.4% [2,25,26]. We demonstrated an 8.5% rate of new postoperative AF, which aligns with the published results of other groups. 

In our study, most patients underwent minimally invasive revascularization via lateral thoracotomy without CPB, aortic clamping, and cardioplegia. Several studies have demonstrated that this approach could lead to a reduction in the level of stroke and neurological complications. These observations might partially explain a total absence of postoperative stroke in our cohort, a feared complication after cardiac surgery. The incidence of stroke typically ranges between 1.1% and 4% in cardiac surgery in general and is estimated around 1.5% in aortocoronary bypass surgery, according to the STS Database update from 2022 [32]. Therefore, this technique could be especially beneficial for patients with severely atherosclerotic or calcified ascending aorta, where cannulation and cross- and side-clamping can increase the risk of serious complications, such as particulate embolism, neurological damage, or aortic injury.

Coronary artery bypass grafting has come a long way since its inception [24,51]. Advancements in technology have transformed the procedure to be less demanding and patient-friendly and to produce repeatable and longer-lasting results [7,10,12,16,24,52]. However, MICS remains challenging for most cardiac surgeons, primarily practiced in high-volume centers by select surgeons [16]. Despite the limited available data, MICS has a more significant benefit in terms of extended survival and fewer postoperative complications than off- and on-pump CABG [37]. Over the years, MICS has proven its value, and the authors believe that improving technology to facilitate visualization, exposure, and reproducibility is still needed to make MICS a standard operational procedure [2,14,25,29,31].

## 6. Study Limitation

The primary limitation of this study pertains to its retrospective design and the absence of control, as it was a non-randomized observational study. The lack of randomization and control may have introduced bias and confounding variables, potentially affecting the validity and generalizability of the findings. Additionally, losing patients to follow up represents a significant drawback, partially attributed to a lack of cooperation and the fact that many patients were referred from external sources. This limitation may have compromised the representativeness of the study population and the accuracy of the conclusions drawn from the data.

## 7. Conclusions

Despite its technicality, MICS is a safe and effective revascularization strategy. It can be successfully performed with low morbidity and mortality and provides excellent long-term results. Our study’s outcomes and the literature suggest that MICS is well suited for patients with isolated LAD disease, patients ideal for hybrid revascularization, and patients with significant comorbidities that preclude MS and CPB, regardless of the urgency of the operation.

## Figures and Tables

**Table 1:** Baseline characteristics. Continuous variables are reported as standard deviation or median (first and third quartiles, IQR), and categorical variables as counts and percentages, n (%). BMI: body mass index; CCS: Canadian Cardiovascular Society; COPD: chronic obstructive pulmonary disease; EuroSCORE II: European System for Cardiac Operative Risk Evaluation II; LVEF: left ventricular ejection fraction; MI: myocardial infarct; NYHA: New York Heart Association; PCI: percutaneous coronary intervention.

Characteristics (n = 315)	N (%)
Age, years, mean ± SD	64 ± 11
Female gender, n (%)	53(16.8%)
Hypertension n (%)	263 (83.5%)
Dyslipidemia, n (%)	280 (88.9%)
Diabetes mellitus, n (%)	94 (29.8%)
Current smoker, n (%)	90 (28.6%)
Ex-smoker, n (%)	109 (34.6%)
Peripheral arterial disease, n (%)	26 (8.3%)
COPD, n (%)	24 (7.6%)
Chronic kidney disease, n (%)	24 (7.6%)
BMI kg/m^2^, median (IQR)	27.1 (24.4–29.8)
Previous cardiac surgeries, n (%)	3 (1%)
Urgent surgery, n (%)	42 (13.3%)
MI in the last 30 days before surgery, n (%)	64 (20.3%)
MI > 30 days before the surgery, n (%)	96 (30.5%)
Previous PCI, n (%)	118 (37.5%)
Preoperative NYHA, n (%)	
I	124 (39.4%)
II	138 (43.8%)
III	46 (14.6%)
IV	2 (0.6%)
Preoperative CCS, n (%)	
0	83 (26.3%)
1	47 (14.9%)
2	109 (34.6%)
3	54 (17.1%)
4	22 (7%)
Number of diseased vessels, n (%)	
One vessel	182 (57.8%)
Two vessels	79 (25.1%)
Three vessels	54 (17.1%)
Preoperative LVEF (%), median (IQR)	60% (50–64%)
Euroscore II, median (IQR)	1.2 (0.8–2.2)
Preoperative Serum creatine (μmol/L), median (IQR)	82 (71–92)

**Table 2:** Operative characteristics. Continuous variables are reported as standard deviation or median (first and third quartiles), and categorical variables as counts and percentages, n (%). CPB: Cardiopulmonary bypass; D: Diagonal branch; GSV Great saphenous vein; LAD: Left anterior descending artery; LIMA: Left internal mammary artery; MIDCAB: Minimally invasive direct coronary artery bypass grafting; MICSCABG Minimally invasive multi-vessel coronary artery bypass grafting; MS: Median sternotomy; RA Radial artery; RCA: Right coronary artery; PI: Pulsatility Index; RIM: Ramus intermedius artery; RIMA: Right internal mammary artery; RCx Ramus circumflex artery. * Patients who required CPB after conversion to MS were omitted.

Intraoperative Details (n = 315)	N (%)
MIDCAB	271 (86%)
MICSCABG	44 (14%)
Duration surgery (min), median (IQR)	139 (115–172)
Pulsatility Index (PI), median (IQR)	1.9 (1.5–2.6)
Graft flow (mL/min), median (IQR)	24 (17–35)
Hybrid revascularization	27 (8.6%)
Conversion to MS	8 (2.5%)
Usage of CPB	12 (3.9%) *
Number of bypasses	
1	254 (80.6%)
2	33 (10.5%)
3	1 (0.3%)
Conduit	
LIMA	313 (99.4%)
RIMA	3 (1%)
GSV	17 (5.4%)
RA	16 (5.1%)
Target vessels	
RIVA	306 (97.1%)
D	27 (8.6%)
RCx	16 (5.1%)
RIM	9 (2.9%)
RCA	3 (1%)

**Table 3:** In-hospital outcomes. Continuous variables are reported as median (first and third quartiles) and categorical variables as counts and percentages, n (%). AF: atrial fibrillation; AV block: atrioventricular block; CK: Creatine kinase; ICU: intensive care unit; IQR: Interquartile range; LVEF: left ventricular ejection fraction; MI: myocardial infarct; PPM: Permanent pacemaker.

In-Hospital Outcomes (n = 307)	N (%)
New AF	26 (8.5%)
Rethoracotomy because of bleeding	5 (1.6%)
New postoperative MI	5 (1.6%)
Postoperative AV block	1 (0.3)
Postoperative wound healing complication	4 (1.3)
PPM Implantation	1 (0.3%)
Postoperative stroke	0
New renal replacement therapy	0
Direct postoperative myoglobin, mcg/L, median (IQR)	63 (30–120)
Highest postoperative myoglobin, mcg/L, median (IQR)	114 (44–8456)
Direct postoperative Troponin T, ng/L, median (IQR)	63 (30–120)
Highest postoperative Troponin T, ng/L, median (IQR)	114 (44–8456)
Direct postoperative CK, U/L, median (IQR)	298 (182–595)
Highest postoperative CK, U/L, median (IQR)	584 (401–838)
Postoperative Serum Creatinine (μmol/L) median (IQR)	82 (72–95)
Postoperative LVEF %, median (IQR)	60% (55–63%)
Duration of hospitalization, d, median (IQR)	8 (7–9)
Duration on ICU, d, median (IQR)	2 (1–3)

## Data Availability

The data presented in this study are available on request from the corresponding author (accurately indicate status).
